# Autophagy and cardiometabolic risk factors

**DOI:** 10.1007/s11154-014-9295-7

**Published:** 2014-09-23

**Authors:** Juan G. Juárez-Rojas, Gissette Reyes-Soffer, Donna Conlon, Henry N. Ginsberg

**Affiliations:** 1 Department of Medicine, Columbia University College of Physicians and Surgeons, New York, NY USA; 2 Endocrinolgy Department, National Institute of Cardiology “Ignacio Chávez”, Mexico City, Mexico; 3 PH10-305, Irving Institute for Clinical and Translational Research, 630 West 168 Street, New York, NY 10032 USA

**Keywords:** Autophagy, Obesity, Dyslipidemia, Inflammation, Insulin resistance, and cardiovascular disease

## Abstract

Autophagy is an essential cellular pathway by which protein aggregates, long-lived proteins, or defective organelles are sequestered in double membrane vesicles and then degraded upon fusion of those vesicles with lysosomes. Although autophagy plays a critical role in maintaining intracellular homeostasis and keeping the cell in a healthy state, this key pathway can become dysregulated in various cardiometabolic disorders, such as; obesity, dyslipidemia, inflammation, and insulin resistance. In these conditions, autophagy may actually worsen the pathological state instead of protecting the cell or organism. In this review, we discuss how dysregulated autophagy may be linked to increases in cardiovascular risk factors, and how manipulation of the autophagic machinery might reduce those risks.

## Introduction

A detailed understanding of how cells maintain homeostasis through the balance of the synthesis and breakdown of proteins is critical to the development of new strategies to prevent and/or treat human disease states. Autophagy, or self-eating, is a general term for the catabolic processes, conserved from yeast to humans, that involves lysosomal degradation of cell constituents (including mitochondria, peroxisomes, and endoplasmic reticulum), unfolded or misfolded proteins, and intracellular pathogens. Autophagy can be induced by a change in cellular environment or in response to starvation when a rapid recycling of fatty acids and amino acids is required to ensure cell survival [1]. Association studies have found a link between autophagy and cardiovascular disease [2–4]; and recently, several publications have reported a strong relationship between autophagy and cardiometabolic risk factors such as obesity [5–7], dyslipidemia [8–10], inflammation [11–13], insulin resistance, and diabetes mellitus [14–16]. The objective of the present review is to describe how dysregulation of autophagy in these states can increase cardiovascular risk, and how manipulation of the autophagic machinery may reverse the disordered metabolism driving these risk factors.

## Autophagy

When first described in 1966, autophagy was considered a non-selective process; however, the findings that different cytosolic proteins are degraded by lysosomes at different rates, and that some organelles or proteins are preferentially degraded under particular conditions, indicate a level of selectivity in lysosome-dependent degradation [17]. Although constitutive mammalian autophagy is essential for basal cellular metabolism and homeostasis, increased levels of lysosomal degradation of cell components have been found under conditions of nutrient or oxygen deprivation, endoplasmic reticulum stress, proteasome malfunction, or damage caused by environment changes [2, 18].

There are three types of autophagy in the cell: macroautophagy (MAC-A), chaperone-mediated autophagy (CMA), and microautophagy (MIC-A). MAC-A is a process in which components of the cytoplasm, including long-lived proteins and organelles, are sequestered inside double-membrane vesicles called autophagosomes. The autophagosomes then fuse with lysosomes to form autophagolysosomes, where the contents are degraded and their molecular components released for recycling or energy production [18, 19]. CMA is driven by the heat shock cognate (HSC) 70, which binds specific polypeptides or proteins containing a KFERQ motif and transports them directly into the lysosome, where interaction with the lysosome-associated membrane protein (LAMP) type 2A receptors leads to protein internalization and degradation [20, 21]. Finally, in MIC-A, defective molecules or organelles are directly engulfed into the lysosomes for degradation and recycling of their components [22]. In this review we will focus on MAC-A.

**Fig. 1:**
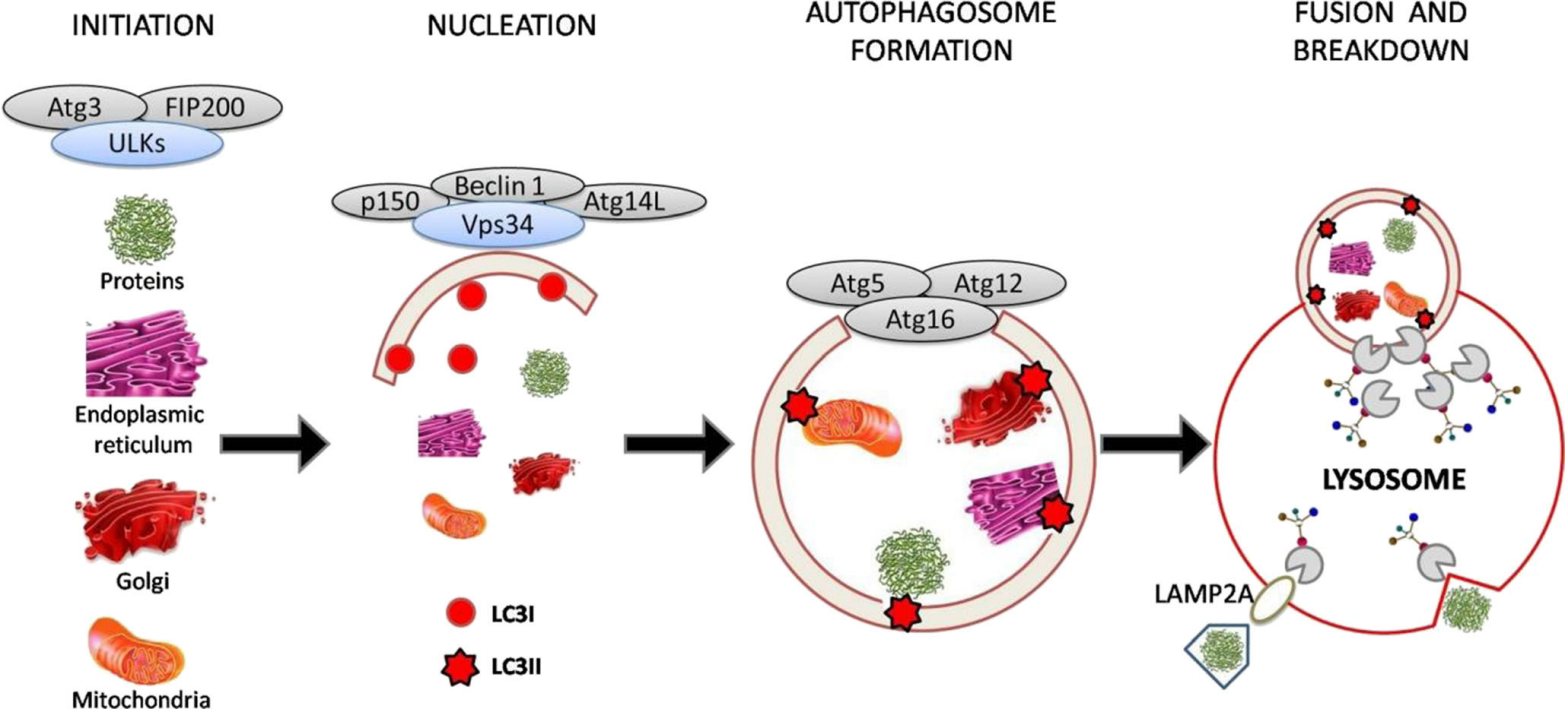
Schematic illustration of autophagy

The MAC-A process is divided in four different mechanistic steps: initiation, nucleation, autophagosome formation, and fusion of the autophagosome with the lysosome resulting in catabolism of the contents (Fig. 1). These processes are mediated by different sets of proteins, encoded by approximately 30 autophagy-related genes (ATGs) characterized in yeast, but highly conserved in eukaryotes cells [4, 18].

**Fig. 2:**
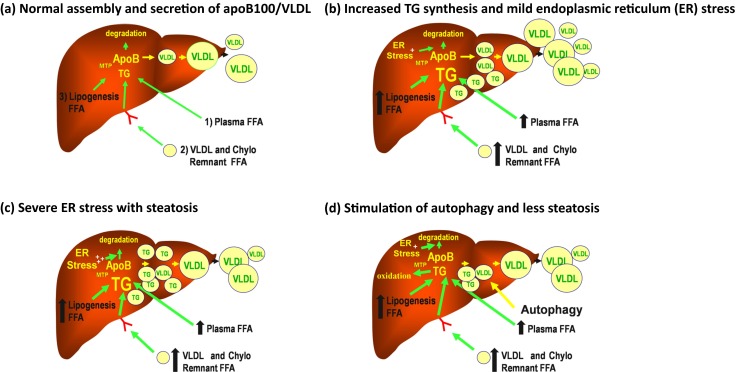
Forces regulating the assembly and secretion of apoB100/VLDL. ApoB100/VLDL is a vehicle for transporting energy from the liver to the periphery and its assembly and secretion are extremely complex. **a** Nascent lipid-poor apoB100 is either targeted for complete translocation into the lumen of the endoplasmic reticulum (ER) for assembly into VLDL or for co- and post-translational ER degradation. The fate of nascent apoB is dependent on lipidation with TG via the action of microsomal triglyceride transfer protein (MTP). The TG originates from free fatty acids (FFA) derived from three sources: 1) lipolysis of adipose tissue, 2) VLDL and chylomicron remnants returning to the liver, and 3) hepatic *de novo* lipogenesis. The VLDL formed in this step then matures with addition of more core lipids, principally TG. This second step is also regulated by MTP and occurs either in the ER or in a post-ER compartment, such as the Golgi. At this point, mature VLDL can be secreted. **b** When TG synthesis from any source is increased, the response is an increase in VLDL secretion. However, mild or moderate ER stress can also result. ER stress can cause increased degradation of apoB leading to less than maximal VLDL secretion and some steatosis. **c** If ER stress becomes even more severe, degradation of apoB increases further and VLDL secretion can fall even more, leading to significant steatosis. **d** Under certain conditions, autophagy can be induced at this point and VLDL can be diverted for post-ER/Golgi degradation. Lipophagy and ER autophagy may also occur, resulting in increased fatty acid oxidation and less steatosis. Thus autophagy might promote both less hepatic steatosis and less apoB100/VLDL secretion

### Initiation

The exact source of the lipids and/or membranes for the initial phagophore is currently unknown and being debated. Some researchers have reported that during initiation, recruitment of lipids from several organelles, depending on the cell type and stimulus, results in the formation of isolation membranes [18]. The initiation step is controlled by a complex of the uncoordinated 51-like kinase 1 (ULK1, also called Atg1), Atg13, and focal adhesion kinase interacting protein of 200 kD (also called Atg17). It is suggested that mammalian Atg13 and Atg17 are phosphorylated by ULK1 [23], and that ULK1 also undergoes autophosphorylation, which is conducive to a conformational change and autophagy induction [24]. Some studies have suggested that Atg13 is phosphorylated by either the mammalian target of rapamycin (mTOR) or ULKs on different residues, which may exert opposite effects on autophagy induction dependent on nutrient status [4]. The mammalian target of rapamycin complex 1 (mTORC1) is an important component of a network that senses the nutrient state of the cell and accordingly controls the levels of anabolism and catabolism to maintain homeostasis [25]. Low glucose levels or high levels of adenosine 5’-monophosphate (AMP), which indicates low cellular energy status, nutrient deprivation, or stress, activate AMP-activated protein kinase, which in turn inhibits mTORC1 activity and induces its dissociation from the ULK1-Atg13-Atg17 complex. As a result, ULK1 is enzymatically active and phosphorylates Atg13 and Atg17 to stimulate the MAC-A process [25, 26]. In contrast, under normal physiological conditions, insulin and insulin like growth factor 1 indirectly induce mTORC1 activity and inhibit autophagy by stimulating class 1 phosphoinositol 3-kinase (PI3K) production of phosphatidylinositol 3-phosphate (PI3P), which induces the Akt kinase at the plasma membrane, and promotes mTORC1 activity [27, 28]. High levels of amino acids also increase mTORC1 activity, by enhancing binding of this complex to regulatory proteins Rag and Rheb (Ras homolog enriched in brain) guanosine triphosphatases [29, 30].

### Nucleation

This step requires a class III PI3K called vacuolar protein sorting 34 (Vps34) that forms a complex with Beclin 1 (also called Atg6/Vp330), p150 (also called Vps15) and Atg14L [31]. Production of PI3P by the Vps34 complex recruits WIPI (WD40 repeat protein interacting with phospho-inositides/Atg18) to the isolation membrane, allowing recruitment of the light chain of the microtubule-associated protein 1 (LC3I/ Atg8) and further maturation of the autophagosome [32]. While specific inhibition of class I PI3K stimulates MAC-A indirectly through downstream Akt/mTOR inhibition, specific inhibition of Vps34 inhibits MAC-A through reduction of an autophagy-specific PI3P pool [33]. The function of Beclin 1 in autophagy has been shown to be regulated by B-cell lymphoma/leukemia-2, an anti-apoptotic protein that inhibits autophagy by binding and sequestering Beclin 1 under nutrient-rich conditions [4]. There are also other mTOR-independent autophagy pathways like c-Jun NH2-terminal kinase 1, which may induce autophagy by phosphorylating B-cell lymphoma/leukemia-2 or Bim (a proapoptotic molecule which inhibits autophagy by its interaction with Beclin 1) and abolishing their inhibitory effects on autophagy [34].

### Autophagosome formation

This step is mediated by two interdependent ubiquitin-like conjugation pathways. In one, Atg12 is activated by Atg7 (E1 activating enzyme) and attached to an internal lysine of the substrate protein Atg5 covalently. Atg12–Atg5 conjugation is constitutive and irreversible and further interacts with a coiled-coil protein Atg16, which allows the Atg12–Atg5–Atg16 complex to become a tetramer by self-oligomerization and also attaches it to the phagophore [4, 33]. The phagophore is an initial sequestering compartment, essential in regulating the membrane elongation and expansion that is essential for autophagosome formation. In the second pathway, LC3I is first cleaved by the Atg4 serine protease and then conjugated to phosphatidylethanolamine by Atg7 and Atg3 (E1 and E2 enzymes, respectively), allowing LC3I to engage the Atg12–Atg5–Atg16 complex on the phagophore. Once fixed to that complex, LC3I is lipidated to form LC3II. The two forms can be distinguished by Western blot analysis. After the autophagosome isolation membrane is formed, the Atg12–Atg5–Atg16 complex is released, whereas LC3II remains associated with the autophagosome until its fusion with the lysosome. In nutrient-rich conditions, the majority of LC3 is cytosolic; upon autophagy induction, LC3 largely exists as LC3II and is localized to both sides of the phagophore [35, 36].

### Vesicle fusion and autophagosome breakdown

The autophagosomes-lysosomes fusion step is mediated by dynein-mediated transport of the autophagosomes along microtubules to fuse with endosomes or lysosomes. When autophagosome formation is completed, LC3II attached to the outer membrane is cleaved from phosphatidylethanolamine by Atg4 and released back to the cytosol [37]. In mammalian cells, the fusion event requires the LAMP type 2A receptor and the small GTPase, Rab7 [38, 39]; however, the exact mechanism remains to be demonstrated. After autophagosome and lysosome fusion, degradation of the inner vesicle is dependent on a series of lysosomal/vacuolar acid hydrolases, such as proteinases A and B (encoded by *PEP4* and *PRB1*, respectively) and cathepsins B, D and L [40]. The resulting small molecules generated from this degradation, particularly amino acids, are released back to the cytosol for re-utilization in protein synthesis and maintenance of cellular functions. The tricarboxylic acid cycle, which utilizes amino acids for generation of bioenergetic molecules and biosynthetic intermediates, appears to co-ordinate with MAC-A through negative feedback of pyruvate on MAC-A [4]. Lipid breakdown supplies free fatty acids needed to sustain cellular rates of mitochondrial β-oxidation and adenosine-5’-triphosphate generation, maintain cellular homeostasis, and promote the metabolism of potentially toxic lipid molecules [10].

## Obesity and autophagy

Increasing rates of overweight and obesity worldwide are closely linked to incidence of metabolic disorders worldwide. Both conditions are associated with accumulation of adipose tissue. White adipose tissue (WAT) functions to store energy in the fed state by taking up free fatty acids and synthesizing TG, which can later be hydrolyzed to provide fatty acids to other tissues during fasting or exercise. In contrast, brown adipose tissue (BAT) has a reduced capacity for lipid storage, but a high rate of β-oxidation that is mediated by the mitochondrial uncoupling protein [10, 41]. Previous reports in cellular and animal models have shown that general obesity and metabolic disease correlate with decreased BAT activity, whereas resistance to metabolic disease from obesity correlates with increased BAT function or the induction of brown adipocyte-like gene expression in WAT [9, 10].

Autophagy has been implicated in cellular development and differentiation including adipogenesis. In a recent study, by Singh and colleagues [5], inhibition of autophagy with 3-methyladenine or knockdown of Atg7 in 3T3-L1 pre-adipocytes reduced accumulation of TG and expression of transcription factors such as peroxisome proliferator-activated receptor gamma and CCAAT-enhancer-binding protein, which are involved in adipocyte differentiation. Inhibition of autophagy was also associated with decreased markers of differentiated adipocytes, such as fatty acid synthase, stearoyl-coenzyme A desaturase 1, and glucose transporter 4. Consistent with these findings, studies in Atg5-null murine embryonic fibroblasts showed a decrease in adipocyte differentiation and lipid accumulation [42]. Additionally, mice with targeted knockout of Atg7 in adipose tissue have decreased WAT mass and increased mitochondrial uncoupling protein and mitochondrial enzymes, leading to a ~32 fold higher β-oxidation in adipose tissue, which suggests that the decrease in lipid storage was secondary to a failure in differentiation and not a direct effect on lipid metabolism [5]. The transformation of WAT from an energy-storing tissue into an energy-expending tissue resulted in a leaner mouse that was resistant to high fat diet-induced obesity, had lower plasma concentrations of free fatty acids, triglycerides and cholesterol, and showed a markedly increased sensitivity to insulin [5, 10]. In line with these results, human studies revealed that autophagy is upregulated in obese individuals, who have increased expression of autophagy-related proteins Atg5, LC3I and LC3II, as well as elevated autophagic flux in both omental and subcutaneous adipose tissue [6, 7]. These results suggest that blocking autophagy in adipose tissue might lead to reduced WAT and increased BAT or beige/browned WAT with greater β-oxidation. Such a change in the balance of WAT and BAT would decrease obesity and some of the associated metabolic disorders [43].

## Lipoprotein metabolism and autophagy

Abnormalities in lipoprotein metabolism leading to dyslipidemias are important in atherogenesis and the development of clinical cardiovascular disease. Lipoproteins are macromolecular spherical complexes of lipids and proteins that carry hydrophobic neutral lipids such as cholesterol esters and triglycerides in a core surrounded by amphipathic phospholipids, free cholesterol, and apolipoproteins. The structure of the lipoprotein allows the hydrophobic core lipids to traverse the hydrophilic environment of plasma. Lipoproteins can be classified according to their hydrated density into chylomicrons, very-low-density-lipoproteins (VLDL), intermediate-density lipoproteins (IDL), low-density lipoproteins (LDL) and high density lipoproteins (HDL) [44, 45]. The role of VLDL (synthesized in liver) and chylomicrons (synthesized in intestine) is to deliver energy, in the form of triglycerides, to peripheral tissues using lipoprotein lipase to generate free fatty acids. After release of fatty acids, the remnants of VLDL and chylomicrons return to the liver where they are taken up to deliver their remaining triglycerides and most of their cholesterol; varying proportions of VLDL remnants and nearly all chylomicron remnants are removed by the liver. Remnant lipoprotein cholesterol plays a key role in the regulation of hepatic cholesterol homeostasis, including regulation of cholesterol synthesis and LDL receptor expression. The VLDL that is not removed by the liver is converted to LDL after passing through the IDL state. VLDL, chylomicron remnants, IDL and LDL, which all carry apolipoprotein B, are each atherogenic because they can permeate the endothelial layer of arteries and initiate and/or increase atherosclerotic plaque formation [10, 45].

Apolipoprotein B100 (apoB100) is a protein synthesized in liver that is essential to the formation of VLDL, IDL and LDL. Considering the critical role that apoB100 plays in lipid transport, metabolic regulation, and atherogenesis, it is not surprising that regulation of apoB100 itself, or apoB100-containing lipoproteins, is complex and occurs at several stages [45]. Multiple apoB100 degradative pathways have been described, and dysregulation of some of these pathways may contribute to the overproduction of VLDL concomitant with increased plasma triglyceride and LDL levels in blood. Factors associated with such dysregulation of hepatic apoB100 metabolism include insulin resistant states such as obesity, metabolic syndrome, and type 2 diabetes mellitus [46, 47].

Like all secreted proteins, apoB100 is synthesized at the surface of the endoplasmic reticulum (ER), where the protein is lipidated during its translocation across the ER membrane by microsomal triglyceride transfer protein (MTP) (Fig. 2). This lipidation allows apoB100 to complete translocation and initiate the assembly of VLDL. In lipid-poor conditions or in absence of MTP, a large proportion of newly synthesized apoB100 is co-translationally ubiquitinylated and degraded by the proteasome. However, some experimental models have shown that approximately 50 % or more apoB100 degradation occurs after translocation, and this is mainly non-proteosomal degradation [45, 48]. The co-localization of proteosomes, autophagosomes, and apoB100 in structures resembling LDs has suggested the involvement of autophagic mechanisms in apoB100 degradation [49]. Furthermore, when the concentration of misfolded proteins rises in the ER, the unfolded protein response is triggered, leading to increased synthesis of ER chaperones and activation of other degradation pathways, including autophagy [45, 50]. Other studies have shown that increased delivery of omega-3 poly-unsaturated fatty acids induce autophagicapoB100 degradation, reducing the secretion of VLDL [51, 52]. The induction of this degradation most likely occurs in the Golgi (post-ER presecretory proteolysis), where the VLDL-apoB100 undergoes aggregation after exposure to lipid peroxides. In contrast to proteosomal degradation, post-ER presecretory proteolysis can occur even when lipid availability and MTP are normal. These data suggest a potential therapeutic role for autophagy in states of increased VLDL secretion and hyperlipidemia. Previous studies have shown that HDL are lipoproteins with antioxidant, anti-apoptotic, and anti-inflammatory properties. A recent study indicated that HDL may also prevent the expression of LC3II and Beclin 1 in human endothelial cells, thereby preventing the autophagy triggered by oxidized LDL [53].

The fact than during fed state, both autophagy and lipolysis are suppressed (leading to cellular lipid storage for future energy demands), and that during periods of limited nutrient supply autophagy increases in parallel with lipolysis, suggest a potential interrelationship between these two pathways. In this respect, very interesting *in vitro* and *in vivo* experiments indicate that inhibition of MAC-A by 3-methyladenine or knockdown of Atg5 or Atg7 genes significantly increased hepatic triglycerides and cholesterol content in mice during prolonged fasting, when delivery of fatty acids to the liver was dramatically increased [7]. The increased hepatic lipid content was present in lipid droplets (LDs), which are cytosolic structures where lipids are stored as a central core of triglycerides and cholesterol esters surrounded by a phospholipid monolayer and associated proteins [54]. Prolonged fasting was also associated with: 1) decreased rates of lipolysis and fatty acid β-oxidation; 2) movement of lipids through the autophagic pathway, indicated by colocalization of neutral lipids with markers of autophagic vacuoles and lysosomes, and by findings of LDs within autophagic vacuoles; and 3) direct interaction of LC3 with LDs before autophagosome formation [7]. These results indicate that autophagy may play a role in the regulation of hepatocyte lipid content, through breakdown of LDs stored triglycerides and cholesterol, by a pathway the authors called lipophagy [9, 10]. This alternative pathway for lipid metabolism may provide hepatocytes with the ability to mobilize large amount of bulk lipids for oxidation when other sources of energy are very limited. These results also suggest that activation of autophagy/lipophagy might be an approach to reducing hepatic steatosis.

## Inflammation, atherosclerosis and autophagy

Inflammation is the first response of the immune system to infection or tissue injury. Although the initial inflammatory response is beneficial, prolonged or chronic inflammation is detrimental, playing a role in diseases such as atherosclerosis [55]. Atherosclerotic lesions begin with dysregulated lipid metabolism resulting in lipoproteins infiltrating the vessel wall, followed by macrophages, leading to the development of lipid-laden foam cells filled with cholesterol ester (CE)-enrich LDs [10, 56]. Breakdown of foam cell CEs by hydrolases is pivotal in the cholesterol mobilization from these cells. In this respect, it has been shown recently that lipid loading activates autophagy in macrophages, thereby targeting LDs for delivery to lysosomes for lipolysis. This allows cholesterol mobilization from LDs for adenosine-5’-triphosphate binding cassette transporter-mediated cholesterol efflux and macrophage reverse cholesterol transport [11]. Importantly, inhibition of macrophage lysosomes only affects cholesterol efflux from lipid-loaded, but not normal cells, indicating a specific role for lysosomal degradation in foam cells [11]. The authors of this study also observed that in lipid-loaded *Atg5* knockout macrophages, cholesterol efflux was decreased, but that inhibition of lysosomal acid lipase had no additional effect on cholesterol efflux, suggesting that decreased autophagic trafficking of CEs to lysosomes was the cause of the decreased cholesterol efflux [11]. Two additional studies support these results and showed that activated macrophage autophagy prevents cellular CE accumulation [14], and promotes resistance to foam cell formation and atherosclerosis [13].

Some *in vitro* studies have also shown that autophagy can be stimulated in atherosclerotic lesions by oxidized lipids [3, 57], reactive oxygen species [58], endoplasmic reticulum stress [59], inflammation [60], and metabolic stress [61]. Although it is possible that basal stimulation of autophagy could be atheroprotective in macrophages, it is also possible that autophagy becomes dysfunctional in more advanced stages of atherosclerosis and loss of autophagic function promotes atherosclerosis, in part, through activation of the inflammasome [62]. Loss of autophagy had been associated with induction of the inflammasome and interleukin-1β processing in macrophages, possibly through either the loss of autophagic suppression of reactive oxygen species accumulation or reduced clearance of molecules that activate the inflammasome [63, 64]. Finally, defective macrophage autophagy has been shown to increases lesion and necrotic area in late atherosclerotic plaques, due to defective efferocytosis of apoptotic plaque macrophages [65]. Autophagy has an important role in the processing of efferocytosed material and has been highlighted as a key mechanism to induce nitric oxide production and reduce reactive oxygen species accumulations [66], which are both also closely related to atherogenesis.

## Insulin resistance/diabetes mellitus and autophagy

Insulin is the major anabolic hormones produced by the pancreatic β cells, and is essential for growth, development, and homeostasis of glucose, fat, and protein metabolism. Binding of insulin to its transmembrane receptor stimulates the intrinsic tyrosine kinase activity of the receptor, which phosphorylates target proteins, such as insulin receptor substrates 1 to 4, to trigger two major kinase cascades, the PI3K and the mitogen-activated protein kinase pathways, that mediate the metabolic and growth-promoting functions of insulin, respectively [67, 68]. Individuals with insulin resistance have increased plasma insulin concentrations resulting mainly from increased insulin production, which is necessary to compensate for resistance to insulin’s actions in hepatic, adipose, and muscle tissues. Insulin resistance is associated with excess body weight, physical inactivity, aging, inflammation, type 2 diabetes mellitus, and cardiovascular disease [69].

The fact that responsiveness to insulin is affected by several processes such as lipid accumulation, reactive oxygen species production, inflammation, ER stress, and mitochondrial turnover, suggest a relationship between insulin response and autophagy pathway. During nutrient deprivation, glucagon causes upregulation of autophagy, whereas excess nutrient supply leads to downregulation of autophagy through insulin signaling [14]. Furthermore, hyperinsulinemic, high fat diet-fed mice [17], as well as obese (ob/ob) mice [18], display impaired hepatic autophagy, as indicated by low levels of LC3II and high levels of p62 (a protein normally degraded via autophagy). Of note, decreased autophagy in ob/ob mice was associated with decreased insulin signaling and increased ER stress, both of which were rescued by Atg7 overexpression [16]. Furthermore, studies in mice with selective deficiency of autophagy in β-cells demonstrated reduced conversion of LC3I to LC3II, marked accumulation of p62, and large protein aggregates in the cytosol of those cells. Importantly, reductions in basal autophagy levels allowed intracellular accumulation of damaged organelles, particularly dysfunctional mitochondria, that promoted increases in reactive oxygen species and reductions in glucose-stimulated insulin secretion in these mice [70, 71], who additionally developed diabetes with marked β-cell loss when fed a high-fat diet [72].

Considering that hyperinsulinemic states decreases autophagy, and that decreased autophagy can, in turn, downregulate insulin signaling, it is clear that autophagy and insulin signaling participate in a feedback mechanism with reciprocal regulation. The molecular basis for this reciprocity is via insulin signaling-mediated activation of mTORC1 during adequate substrate availability and mTORC1 inhibition of insulin signaling during decreased substrate availability [25, 28]. Although autophagy also may protect β-cell function [73], accelerated autophagy also appears to be involved in β-cell death under special conditions [67].

## Potential targets in the autophagic machinery

Most of the results previously discussed suggest that regulation of autophagy could be a useful target to prevent or control some of the metabolic abnormalities associated with cardiovascular disease or atherosclerosis itself (Table 1). For example, experimental studies have shown that blocking pathways that stimulate autophagy, particularly in adipose tissue, may reduce WAT and increase β-oxidation; which in turn would decrease obesity and the associated metabolic disorders. It is important to highlight that any attempt to inhibit autophagy to prevent or treat obesity must first address important questions as to how such inhibition might affect mitophagy (process to remove damaged mitochondria through autophagy) in mature adipose tissue, as well as what would be the systemic effects of this inhibition [9, 74]. Elucidating the answers to these questions could provide the basis for novel approaches to converting WAT to BAT and effectively treating obesity and its associated metabolic disorders [43]. Conversely, activators of autophagy such as rapamycin and its analogs, everolimus and sirolimus, which act via mTOR inhibition, have shown promise, in preclinical studies, as beneficial potential therapeutics for coronary heart disease. These mTOR inhibitors, routinely used in drug eluting stents, were found to prevent atherosclerosis development and progression in atherogenic mouse models [75, 76], as well as specifically reduce the cholesterol content of the aortic arch [77]. Furthermore, a selective clearance of lesional macrophages and rise in apolipoprotein A1 and HDL were observed after rapamycin or sirolimus treatment, which suggests another plausible mechanism by which mTOR inhibition exerts atheroprotective effects [78, 79]. Although pharmacological inhibition of mTOR can increase plasma levels of LDL-cholesterol and triglycerides [80], as well as proinflammatory cytokines such as interleukin-6, monocyte chemoattractant protein-1, and tumor necrosis factor-α [81], combined treatment with lipid-lowering therapy or anti-inflammatory agents, may help to prevent the adverse effects of drug-induced autophagy without affecting the ability of these agents to deplete lipids and reduce macrophages with in atherosclerotic plaques [80, 81].

**Table 1:** Effects of modifying autophagy on cardiovascular risk factors

Cardiovascular		
Risk factor	Potential target	Effect
Obesity	Decrease autophagy	↓ Triglycerides in plasma ↓ Cholesterol in plasma ↓ Lipid store ↑ Free fatty acid β-oxidation ↑ Obesity resistance
Dyslipidemia	Increase autophagy	↓ Triglycerides in plasma ↓ Cholesterol in plasma ↓ Lipid store ↓ Low density lipoprotein oxidation ↑ Free fatty acid β-oxidation ↑ Folding and traffic proteins
Inflammation	Increase autophagy	↓ Inflammasome ↓ Foam cell formation ↓ Cholesterol ester acummulation ↓ Reactive oxygen species ↑ Nitric oxide ↑ Transport reverse cholesterol ↑ Lipophagy
Insulin Resistance	Increase autophagy	↓ Damaged organelles ↓ Mitochondrial dysfunction ↓ Reactive oxygen species ↑ Endoplasmic reticulum stress ↑ Glucose stimulated insulin secretion ↑ Insulin sensitivity

## Conclusions

Autophagy is primarily a protective process that plays a role in recycling cellular constituents and maintaining cell homeostasis. Based on the literature reviewed here-in, it appears possible that reducing autophagy in adipose tissue might be an approach to controlling obesity whereas increasing autophagy in the liver, β cell, or atherosclerotic lesion might provide protection from hyperlipidemia, diabetes mellitus, and coronary events secondary to rupture of necrotic atherosclerotic plaques. However, developing tissue specific approaches with narrow therapeutic benefit:risk ratios will be very challenging.
